# Workshop report: Everyday environmental heritage is key to unlocking the social dimensions of One Health

**DOI:** 10.1016/j.isci.2025.112358

**Published:** 2025-04-18

**Authors:** Marco J. Haenssgen, Tania Angeloff, Mark R. Herse, Elizabeth Auclair, Navaporn Sunanlikanon, Thipphaphone Xayavong, Jean-Marc Dubost, Somphavanh Radavanh, Chantho Vongnalath, Eric Deharo

**Affiliations:** 1 Department of Social Science and Development, Chiang Mai University, Chiang Mai, Thailand; 2 UMR Développement et Sociétés, Université Paris 1 Panthéon-Sorbonne, Paris, France; 3 Conservation Ecology Group, King Mongkut’s University of Technology Thonburi, Bangkok, Thailand; 4 Department of Geography, CY Cergy Paris Université, Cergy, France; 5 Global Sustainable Development, University of Warwick, Coventry, UK; 6 Independent Researcher, Rectorat de Versailles, Versailles, France; 7 Biosafety Division, Department of Science, Ministry of Education and Sports, Vientiane, Lao PDR; 8 Village Chief, Ban Hat Khay Village, Bolikhamsai Province, Lao PDR; 9 UMR MIVEGEC, French National Research Institute for Sustainable Development (IRD), Montpellier, France

## Abstract

International health organizations and experts and researchers from diverse fields consider the “One Health” concept (interdependence of human, animal, and environmental health) key to understanding global health security challenges such as the risk of pandemics from zoonotic disease emergence. However, the knowledge surrounding One Health retains critical blind spots for effective intervention design which we seek to address in this backstory article. We foreground lessons learned from an international workshop on everyday environmental heritage, held in Lao PDR in December 2024, to demonstrate the value of interdisciplinary and participatory exchanges for advancing the frontier of One Health scholarship and practice.

Despite the salience of the concept and its deep historical roots, social factors that influence the local meanings of—and interactions between—humans, animals, and the environment remain poorly understood in One Health scholarship.”

Everyday environmental heritage is by definition intrinsically valued by community members, but it is also dynamic, continuously changing, and it can attain political qualities if it responds to environment-related threats that a community is experiencing.

Environmental heritage can be a powerful reason and means for practicing conservation, whereas policies that alienate local communities and restrict heritage opportunities can undermine conservation by eroding culturally important incentives to defend or restore ecosystems.

Systematic exploration aided by participatory methodologies can provide a place-based understanding of social realities that is sensitive to gender dynamics and various forms of intersectional disadvantage, without romanticizing or demonizing human behaviors.

Participatory or ‘bottom-up’ approaches to One Health are currently constrained by contemporary systems of environmental and economic governance and restrictions on human rights to self-determination.

## Main text

### Workshop motivation

One Health is a concept that recognizes the interdependence of human, animal, and environmental health. This idea holds central importance for improving global health security through its integrated understanding of international and local challenges such as the risk of pandemics from zoonotic disease emergence, antimicrobial resistance, and food insecurity through environmental degradation and contamination. The concept was first formally introduced in 2004 at a conference of the World Conservation Society, although its core principle of interdependence had already been recognized by many Indigenous peoples for millennia. Despite the salience of the concept and its deep historical roots, social factors that influence the local meanings of—and interactions between—humans, animals, and the environment remain poorly understood in One Health scholarship. For example, worship of natural deities, burial traditions, gendered ritual practices, traditional pastoralism and hunting, and rural development processes can shape land use patterns, ecological communities and habitats, and interactions that people have with animals and the environment.^1^

This black box is not merely an academic challenge; lacking a grounded understanding of peoples’ lived realities and methodological toolkits to assess them can leave policy makers to rely on technocratic solutions designed and implemented from the top down. A typical example is the awareness raising approach (e.g., in environmental education campaigns), which presumes that seemingly problematic human practices stem merely from a deficit of knowledge that can only be rectified through education activities imparted by external experts. Such top-down formulations can be both counterproductive and unjust, as has been seen, for instance, in global forest conservation efforts that have exacerbated forest loss and degradation by displacing Indigenous peoples who have key roles in forest management and conservation all over the world.^2^^,^^3^

**Figure:**
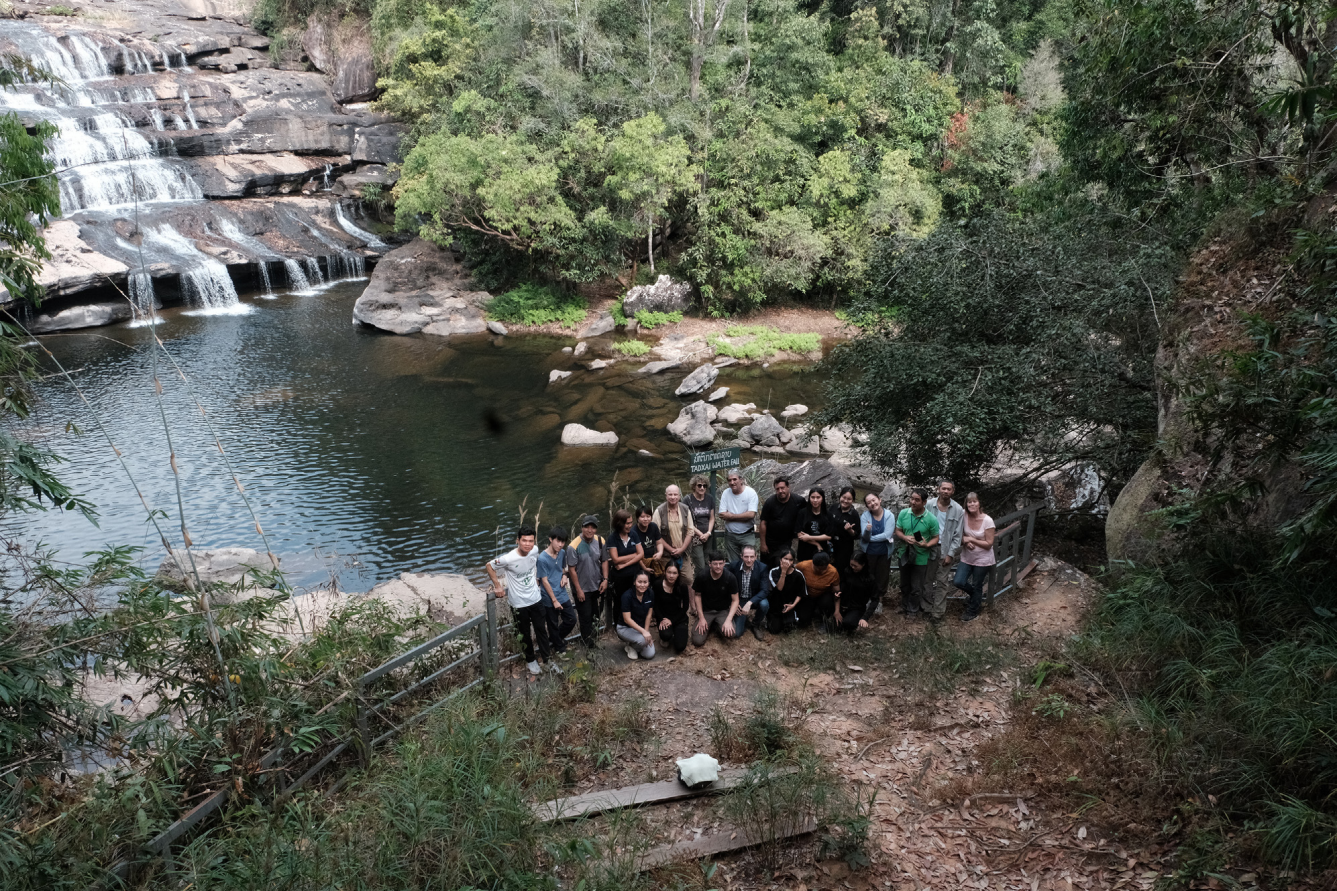
Workshop participants gather during an excursion at Tad Xai waterfall for a group photograph. Source: Authors (M.J.H.), presented with media consent of all depicted individuals.

Many scholars and policy makers understand this problem and have increasingly advocated for participatory bottom-up approaches that respect customary rights, institutions, and systems of knowledge.^1^ However, proposals for how to concretely and systematically realize such alternative approaches are still lacking. International scholars therefore convened a participatory workshop in Lao PDR in December 2024 to develop and apply the concept of everyday environmental heritage to advance the social considerations of One Health. The 4-day workshop involved interdisciplinary conceptual contributions to studying the interface of heritage and One Health, methodologies to capture and assess One Health in a grounded and participatory fashion, and the application thereof in case studies and in the local context of a community situated at the border of Phou Khao Khouay National Park in northern Lao PDR. The 20 workshop participants included Lao, Thai, and Western scholars from different backgrounds, Lao academics, and Lao students. Members of the local community joined as workshop contributors and local guides, thus helping to tackle concepts in consultation—including through community members’ own spatial representations of their village. The participatory design of the workshop enabled the attendees to explore heritage and One Health in their village and through their eyes, and to jointly reflect on directions for One Health policy. In this backstory, we present the lessons learned from this participatory and interdisciplinary engagement.

### Conceptual approaches to heritage and One Health

#### Ordinary heritage in the context of global sustainability

The workshop was opened by E.A. with foundational explorations of key concepts, including “heritage”. International and national bodies have started to recognize the importance of culture and heritage for fostering sustainability – and, by implication, One Health as an important domain of sustainable development. Culture is thereby frequently seen as a major component of sustainability, either as its “fourth pillar” or as a transversal dimension, and the concept of heritage both forms an intuitive subset of culture and fulfills important functions in supporting as well as transmitting culture. However, contrary to a focus on culture, the concept of heritage helps raise contemporary issues of preservation and conservation as well as of creation, adaptation, and resilience to changes in the sphere of sustainability.

Using heritage as lever for contemporary sustainability policy is the outcome of a long evolution, which is partly captured by the development of major international texts such as the 1972 UNESCO Convention on Cultural and Natural Heritage, the 1992 UNESCO category “living cultural landscape”, the 2003 UNESCO Convention on Intangible Heritage, the 2005 UNESCO Convention on the Protection and Promotion of the Diversity of Cultural Expressions, or the 2011 UNESCO Recommendation on Historic Urban Landscapes.^4^ This development has involved broadening the concept of heritage and articulating tangible and intangible heritage; acknowledging the diversity of personal, family and community identities, memories and histories; and encouraging dialogue between generations, between neighborhoods and between communities.^5^ Heritage has come to be understood as a social process that links the past, the present, and the future. This development also implies taking into account “ordinary” heritage, which is the “everyday” and “living” heritage related to and encountered in daily practices.^5^

Based on this conceptual starting point, the workshop participants discussed underlying principles and assumptions of heritage to enable consistent engagement with the social aspects of One Health. First, this involved articulating three cross-cutting dimensions that operationalize the concept of ordinary heritage as (1) a people-centered process, (2) a place-based approach, and (3) a method fostering citizenship and local democracy.

1. **A people-centered process:** Ordinary heritage reflects a social process and not merely a collection of objects to preserve. Even though economic goals can be considered, heritage can be a crucial lever for social inclusion and environmental preservation. This means recognizing the inhabitants as well as visitors and tourists.
2. **A place-based approach:** Consistent dealing with ordinary heritage will entail bottom-up methodologies in which the values of proximity, closeness, nearness, and localness are fortified. These values can foster attachment, sense of place, and sense of belonging amongst the inhabitants. Practical considerations for approaching ordinary heritage in research and policy thus include transversality (linking heritage with culture, education, social action, health, ecology, planning, etc.), networking (relations with other communities or sites to prevent cultural isolationism), and the multi-scale articulation of heritage in national, local, and Indigenous cultures.
3. **A method for fostering citizenship and local democracy:** Ordinary heritage invites action research approaches that help create enabling conditions for the participation of local inhabitants in defining their heritage, which in turn leads to a better understanding of place and the local environment. However, such can only be achieved by fostering empowerment and reinforcing the capabilities of the inhabitants.

Ordinary heritage defined as such aligns with political movements fighting for social justice, local democracy, and greater environmental awareness. Implicit in this idea is a high level of trust in people to share and protect their common resources, which resonates with alternative visions for sustainable development that de-emphasize individualistic attitudes of consumers pursuing their own interests.^6^ This approach to heritage offers an innovative pathway to redefine the roles of the different actors involved in governance (state bodies, local governments, private stakeholders, experts, civil society, NGOs, activists, local inhabitants, etc.), with greater emphasis on transdisciplinary approaches that combine ecology, architecture, urban planning, heritage studies, art and other disciplines, and toward developing environmental projects that de-emphasize individual consumption and private property.

#### Everyday environmental heritage and its links to One Health

While ordinary heritage generally has strong links to sustainable development, the workshop developed further the intricate links between heritage, environmental considerations, and One Health. Everyday environmental heritage emerged as key in linking these domains, forming a subset of ordinary heritage that inherits all its defining features. The “environmental” aspects relate the heritage directly to people’s local ecosystem – as a component of heritage practices and objects (e.g., using natural materials), as object (e.g., if practices are directed at natural deities or convey environmental values), as site or location where the practice takes place (e.g., rituals in a forest or at a spring), or instances that otherwise create a “sense of belonging and attachment” toward the natural environment. The “everyday” aspect helps challenge probable biases that equate heritage with “traditional” practices because it can also pertain to modern and contemporaneous issues like changing ecosystems, marketing natural products by forest-dwelling communities, or changing landscapes. The process character to enable these expressions of heritage also helps avoid romanticization because community members need to continually negotiate what practices are worth preserving (as their views might diverge even within a community), hold meaning for them, and whether or how they are benefiting their local ecosystems. The resulting everyday environmental heritage is by definition intrinsically valued by community members, but it is also dynamic, continuously changing, and it can attain political qualities if it responds to environment-related threats that a community is experiencing.^2^

While everyday environmental heritage is an anthropocentric concept focused on human practices, M.J.H. explored how it may link to the various elements of local One Health systems. We demonstrate in the case studies below how these links materialize concretely, but generic examples are illustrated in teh summary diagram below and include links to:

1. Human health: As a sense of belonging and attachment, everyday environmental heritage fulfills important functions of social and spiritual health that are important to human well-being – to the extent that people may sacrifice their physical health for it. Likewise, knowledge and usage of medicinal herbs can have a direct bearing on human health and indirect bearing even on global health issues. For example, local trends that favor the use of antibiotics for self-medication over traditional pharmacopoeia remedies may play a role in the emergence of antimicrobial-resistant pathogens.
2. Environmental health: Environmental heritage practices can directly contribute to the conservation of forest spaces, for instance, by declaring sacred and community forests with ritualistic importance.^2^ Indirectly, human presence in forests through heritage practices also enables them to detect forest fires or otherwise manage lands to limit accumulating fire risks. However, heritage practices do not by definition protect local ecosystems; while some practices can be beneficial, the evolution of others (e.g., eco-tourism or artisanal fishing) can also potentially create environmental damage (however, the dynamic and process character of heritage allows that such aspects can evolve and be negotiated in community members’ recognition of what heritage is).
3. Animal health: Environmental heritage practices also extend into food preferences and the related aspects of hunting and food preparation practices.^1^ Hunting and other engagements with local ecosystems also shape patterns of human-animal encounters and thus the risk of zoonotic spillovers.

In turn, interactions among these elements create second- and third-order One Health impacts driven by heritage – for instance, forest fire risks influence wildlife habitats and migration, and in turn human-animal encounters and the likelihood of zoonotic spillovers as well as the food security situation of local communities.

**Figure:**
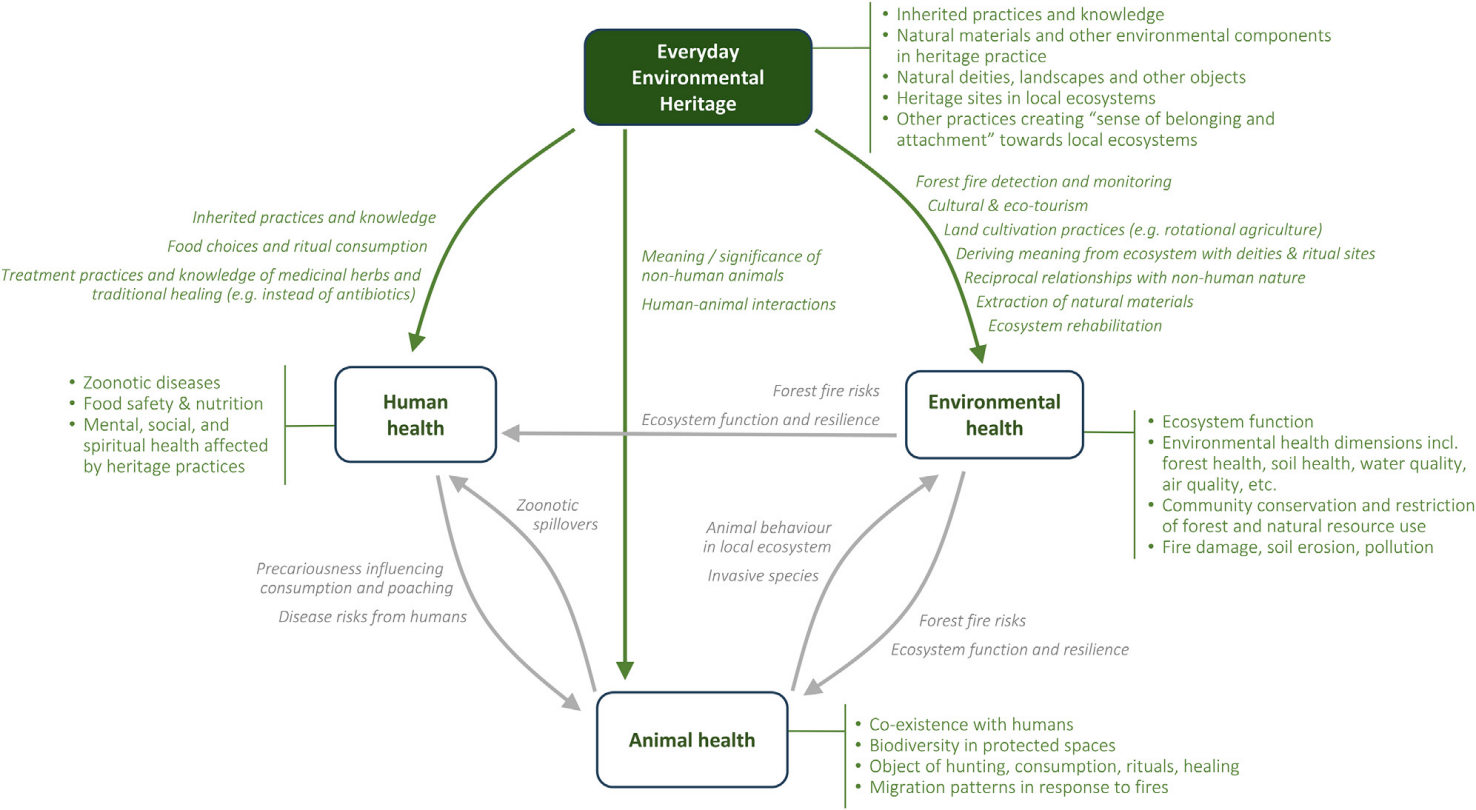
Illustrative linkages between everyday environmental heritage and One Health components. Notes: Non-exhaustive list for illustration only. Note that linkages do not imply that connections and pathways are virtuous, e.g., eco-tourism and bushmeat hunting can have varied impacts on ecosystems.

Social justice objectives of ordinary heritage come again to the fore in the operationalization of these considerations for One Health and environmental policy. A key issue raised by T.A. is the gendered dimension of inequalities – notably, the unequal use of natural resources between men and women, ethnic minorities and more dominant groups, and so forth. Despite the policy of “gender mainstreaming” being promoted at all institutional levels since 1995 (UN Beijing Conference), gender-specific approaches and intersectional perspectives remain minor in the analyses underpinning policy decisions.

One concept in particular can be used to reactivate considerations of intersectionality in heritage-sensitive development and environmental policy: “environmental care”. This concept explicitly recognizes the potential harms that can arise from purely top-down approaches to environmental governance. It advocates taking account of marginalized communities in the protection of nature based on ethics rather than on vertically imposed technical measures.^7^ In this sense, it is an intersectional approach to environmental issues, which intertwines gender, class, and race inequalities. Environmental care is based on the definition of care by Tronto and Fisher^8^ as “a species of activities that includes everything we do to maintain, contain, and repair our ‘world’ so that we can live in it as well as possible.” When applied to the environment, the ethic of care emphasizes individual and collective responsibility, interdependency between all species and with our environment, and a need to take concrete action far beyond philosophical reflection. Last but not least, environmental care enables us to move away from a purely anthropocentric analysis toward reason “beyond [the opposition between] nature and culture”.^9^ Through the ethics of environmental responsibility, this concept enables us to question and redefine what “nature” and the “natural environment” are, to question the place of every living being and ecosystem, while encompassing the interdependent relationships between humans and non-humans.

### Heritage and One Health in action

#### Big-headed turtles

To illustrate the importance of environmental heritage in determining One Health outcomes, M.R.H. drew on a case study of critically endangered big-headed turtles (*Platysternon megacephalum*) and a rural Khon Muang community’s river conservation initiative in northern Thailand. The community was situated along a national park boundary where villagers were permitted by the Thai government to live and cultivate lands legally owned by the state. Such ambiguous state-owned lands have been widespread in Thailand and degraded in many places by commercial agriculture, state-sponsored development projects (e.g., logging, dams), and forest conservation policies that undermine customary systems of management.^2^^,^^3^ In this case, the Thai government and private development companies have planned to build a dam near the village, which many local villagers opposed because it would destroy a sacred site, inundate cultivated lands, and threaten the local fishery.

Fishing has been a locally common environmental heritage practice for procuring preferred foods and attaining recreation and solitude. Fishing has also been instrumental in fostering a sense of attachment to place and community through annual fishing festivals, and production of ecological knowledge through regular engagement with the river ecosystem. Although villagers have established a community-protected area near the village to control potential overfishing and preserve their fishing heritage, they have been limited in their ability to prevent the planned dam project due to restricted land and decision-making rights. Considering these limitations, concerned villagers have partnered with researchers and the grassroots NGO Living River Association to strategically leverage an extant population of big-headed turtles to oppose the dam project, while promoting turtle conservation within their social networks of fishers and hunters. These villagers, including local fishers and hunters who are regarded by some external experts as threats to nature, have essentially been serving as a last line of defense for a critically endangered species and river ecosystem. The overarching lesson from the case was that environmental heritage can be a powerful reason and means for practicing conservation, whereas policies that alienate local communities and restrict heritage opportunities can undermine conservation by eroding culturally important incentives to defend or restore ecosystems.

Discussions of this case during the workshop demonstrated how heritage and One Health considerations resonated with Lao academics, students, and village leaders. Lao villagers at the workshop shared their experiences regarding a dam project near their community, noting its impact on the local fishery and freshwater ecosystem, along with limited economic benefits for the area. The village leader emphasized the importance of empowering local decision-making to address environmental concerns effectively and suggested that adjustments in economic practices could help alleviate pressures on ecosystems and households. Furthermore, Lao students expressed a desire for more opportunities to collaborate with local communities and policymakers to support positive change.

#### Multispecies heritage

The second case study, presented by J.-M.D., related to the interdependencies of two species in everyday environmental heritage as it interrogated the interactions and multidirectional knowledge flows between mahouts (elephant caretakers) and elephants in Lao PDR in relation to the production of medicinal practices. When addressing heritage issues, the production of medicinal knowledge on the use of natural resources to maintain good health and cure health disorders is often implicitly understood as a human prerogative. However, a growing body of ethological studies has shown that non-human animals also use natural resources to cure themselves and maintain their health, giving way to the emergence of a new research discipline, zoopharmacognosy, which focuses on animal self-medication behavior and the medicinal properties of the substances that animals use for healing or prophylactic purposes (note that we use the term “animals” for convenience to refer specifically to non-human animals). In parallel, numerous anecdotes drawn from oral traditions attribute the origin of various human medicinal uses of plants to animals’ self-medication behavior, the memory of which has often been preserved in their vernacular names.

J.-M.D.’s studies highlighted a set of convergences between elephant self-medication observations as reported by mahouts, and their own medicinal practices.^10^ Alongside plants from the local human pharmacopeia, mahouts have been using plants they saw elephants seek out when ill. The mahouts’ medicinal use of some plant items in their households also appeared to be more consistent with their observation of elephants’ self-medication behavior than with the use of the same items by local healers, which strongly suggested transfers of medicinal knowledge from elephants to humans.^11^ Conversely, as mahouts treated their elephants with plants from the local human pharmacopoeia, collected in an environment shared by both species (as their elephants fed themselves in the surrounding forests), a potential transfer of medicinal knowledge from mahouts to elephants could not be ruled out either.

These interdependencies highlight the complexities of everyday environmental heritage, as it is, for instance, difficult to determine whether knowledge and practices are of human or animal origin. It rather becomes more relevant to approach this medicinal knowledge as a set of a multispecies knowledge whose construction results from multiple interactions involving human and non-human actors, a view that falls within the concepts of hybrid communities and multi-specific cultures considered and studied as a whole.^12^ This medicinal knowledge is therefore best understood as part of a multispecies heritage,^11^ which is very much in line with a One Health perspective, as it directly affects the health of both humans and non-human animals. Preserving this type of intangible heritage related to interspecific uses of shared natural resources requires maintaining access to those resources for all individuals (human and animal) involved in the co-construction of knowledge linked to their use. It also requires preserving the ecosystems that produce them. This dual requirement challenges conservation policies that are often based on the exclusion of human activities in protected areas.

### Methodological approaches for heritage in a One Health setting

#### Participatory mapping

To make heritage in One Health systems visible, T.X. formulated a practical way of eliciting local knowledge on heritage practices and their environmental dimensions. Following welcoming remarks and introductions from the village committee, a mind-mapping activity was introduced to the meeting participants as a tool to learn more about everyday heritage in their village.

Workshop participants and the community representatives were divided into three small groups of five to eight people to foster active engagement and ensure equal opportunities to participate among all attendees. Methodologically, the participatory component of this exercise relates especially to the process of learning and knowledge co-production. Power relations between the community representatives and workshop participants were acknowledged and deliberately negotiated during this exercise.^13^ While the map drawing served as a visualization tool, it also created a space where the villagers took a more active role in choosing and presenting their heritage, knowledge, and stories – which facilitated the equilibrium of power relations between the workshop participants and the community members. Given that the positionality of the workshop participants were academic scholars and students in higher education, our learning of everyday heritage did not take the conventional (research) approaches of interviewing or asking questions where the community members are treated as merely informants. Instead, the role of cultural heritage bearers and practitioners of the community members was emphasized to lead this learning process.

**Figure:**
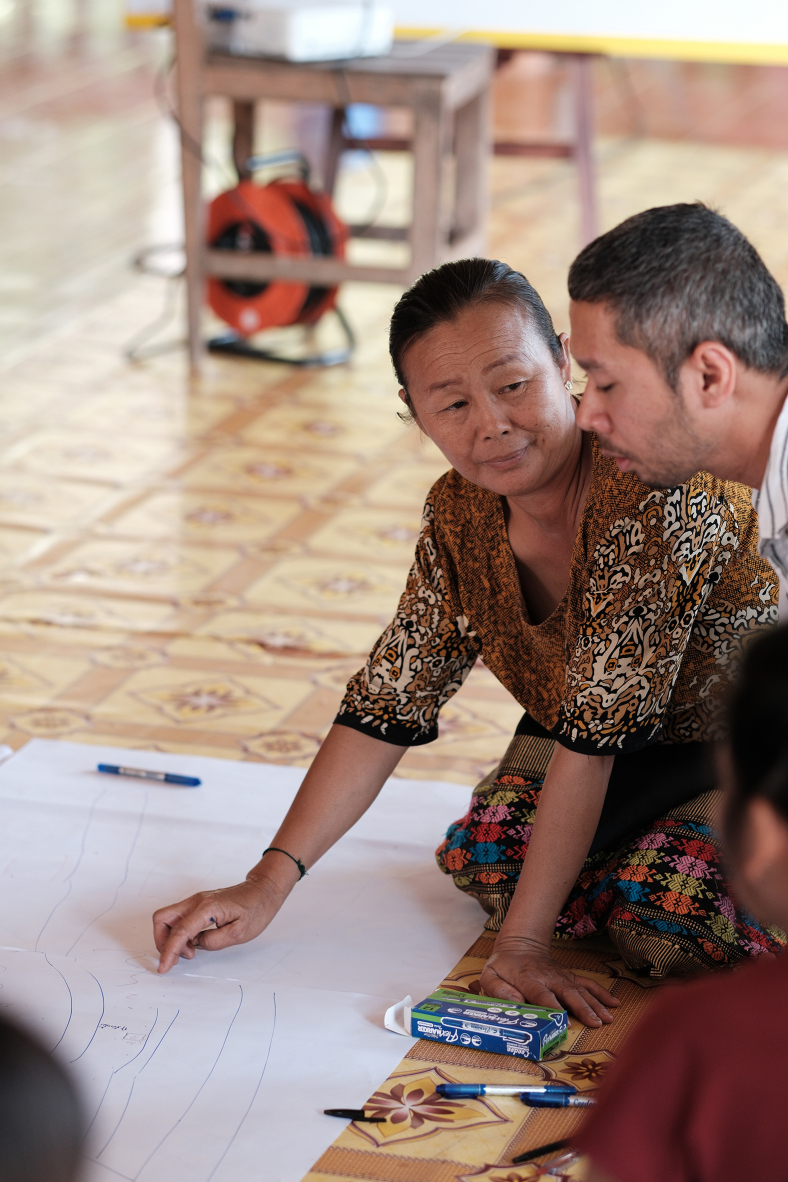
A villager explains the location of local heritage sites in her community to the session facilitator. Source: Authors (M.J.H.), presented with media consent of all depicted individuals.

During this activity, aspects of everyday heritage of the village were highlighted and visualized on the maps, for example, representing the locations where medicinal plants were preserved and harvested, the spiritual boundary of the village, its sacred sites, and more. These geographical features not only represented local knowledge and cosmology but also demonstrated how some everyday health practices of the people continued to rely on the interdependent relationship between humans and local ecosystems, which has been maintained through the continuation of their everyday heritage. This methodological demonstration also highlighted that the study of everyday heritage has the potential to be further utilized to develop appropriate tools and methodologies for researchers to *learn from* local knowledge and practices, particularly those related to One Health, which have existed but remained underrepresented due to the lack of suitable methodological approaches.

#### Systematic scoring

Despite their apparent complexity, it is also possible to provide systematic assessments to select and promote promising instances of heritage for One Health policy – building on the concepts and underlying principles discussed at the workshop. A case study presented by N.S. drew on the experiences of four Indigenous communities in Chiang Mai Province, Thailand, and highlighted how a practical scoring method embedded in a participatory approach can be employed.

Firstly, as outlined above, participatory methods need to establish the range of everyday environmental heritage instances as well as their recognition and role in a community. In the case of Chiang Mai, tools like the *Community map* helped local ethnographers in each community to familiarize themselves with key locations and heritage-related activities, followed by the development of a *Social structure map* to reveal power dynamics among leaders and villagers – thus guiding ethnographers in navigating community relationships and avoiding conflicts. In addition, an *Annual calendar* was compiled to highlight temporal and spatial patterns of everyday environmental heritage by exploring agricultural cycles, cultural events or festivals, and events and gatherings with collaborating organizations external to the community. The information from these tools fed into a *Heritage record form*, which provided a “thick description” (i.e., richly contextualized characterization) of salient heritage elements across five categories: connection with ecosystems, way of life, identity, power negotiation, and public engagement. The local ethnographers identified five to ten relevant instances of heritage in each community, focusing on both cultural and environmental dimensions. Subsequently, the research team used an *Evaluation form* comprising a checklist and questionnaire to assess these heritage elements’ relevance to community life and their a-/political significance. Three to five key forms of heritage were then selected by the team for in-depth analysis based on their highest scores and local relevance. To facilitate the ongoing conversations with the communities, full-text *Preliminary analysis summaries* further evaluated each instance of heritage for its political and instrumental roles in promoting human security and informing conservation policy. On this extensive qualitative and semi-quantitative data basis, in each community the research team selected two instances of high-potential heritage for broader consultations with community members and representatives to gauge their possible policy viability and implications. The consultations were aided by the production of leaflets and large banners in English, Thai, and the local languages Hmong and Karen. The resulting selection of heritage practices for further promotion on the community level (e.g., tree ordination in one of the communities) ensured that policy action would not only be locally grounded but also legitimate and acceptable to the participating communities.

In summary, the systematic scoring approach demonstrated an effective way to assess everyday environmental heritage and its implications for policy advocacy. The case study highlighted the critical role of Indigenous practices in linking cultural heritage, environmental sustainability, and public health. By combining systematic and participatory ethnographic methods, the heritage scoring approach can generate policy-relevant outputs that are sensitive to the place-based and local democracy approaches and gender dynamics, which are crucial principles of everyday environmental heritage.

**Figure:**
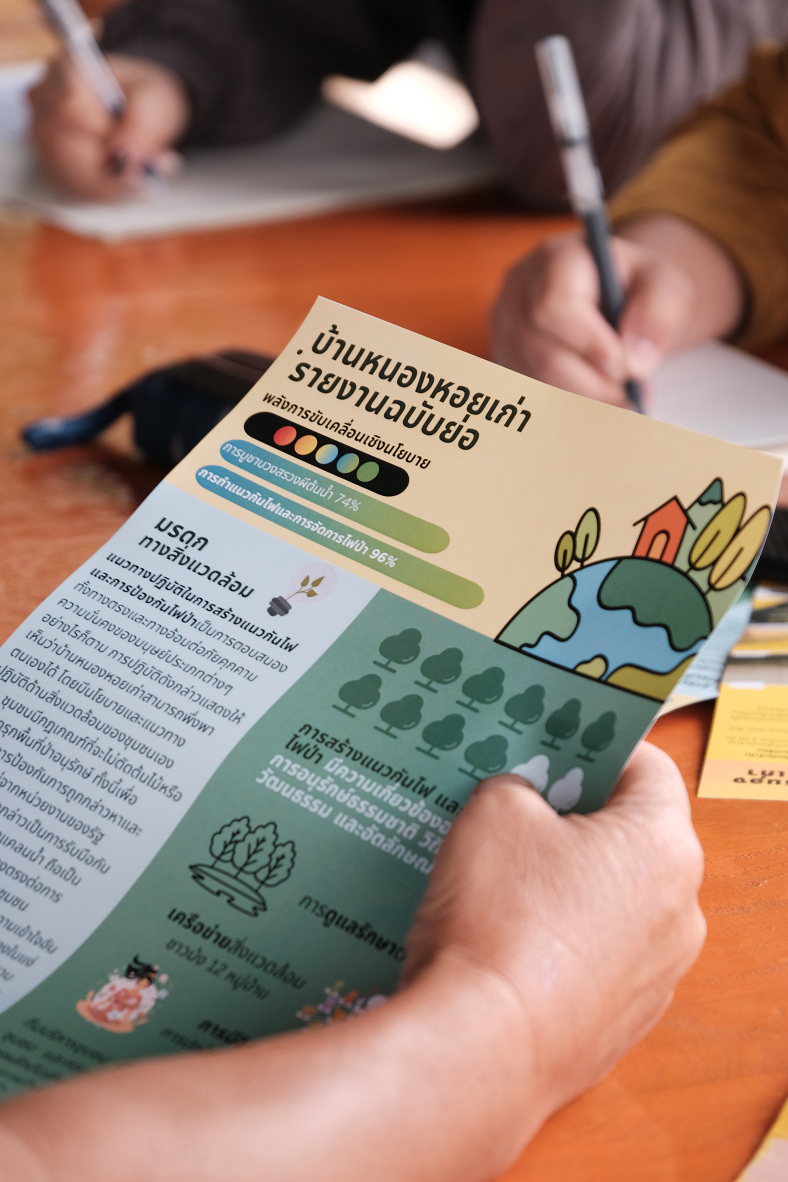
A village representative in Chiang Mai considers suggestions for heritage interventions presented on a leaflet. Source: Authors (M.J.H.).

### Final reflections from the workshop

The workshop discussions and exchanges consistently reiterated the relevance of everyday environmental heritage for understanding academic and policy challenges that currently limit the potential of One Health to alleviate health security risks. The insights were enabled by a workshop with participants from diverse backgrounds, and constructive interactions with trusted intermediaries who established links among village community representatives, students, and researchers. Moreover, joint activities to map, interpret, and visit local spaces fostered mutual interest and enabled all participants to make observations that conventional interviews would not have brought to light. Likewise, the workshop demonstrated that systematic exploration aided by participatory methodologies can provide a place-based understanding of social realities that is sensitive to gender dynamics and various forms of intersectional disadvantage, without romanticizing or demonizing human behaviors.

The dynamic nature of environmental heritage makes it a powerful basis for engagement and negotiations between different workshop participants. However, from a process perspective, it is important to interrogate the academic value of such events, and question the extent to which such events could benefit or undermine participating communities, to avoid unintended consequences. For instance, our discussions and case study experiences highlighted that village elders may feel reluctant to share their cultural knowledge, especially knowledge of traditional medicine. Specifically, some traditional medicine practitioners may feel that their knowledge should only be transmitted within their respective families to preserve the legacy of their family. Similarly, some communities may safeguard the integrity of their heritage and knowledge by withholding information from outsiders. In other cases, the sharing of knowledge may be more nuanced. For instance, in the case study of a Hmong community in Chiang Mai, elders had become increasingly aware of the risk of their knowledge being lost entirely due to younger generations’ declining interest in traditional practices as illustrated by the shift from traditional medicinal practices to the widespread use of biomedicine remedies. Consequently, some elders have become more willing to share their knowledge outside of their respective families and communities, if learners agree to never perform the rituals themselves. Thus, we stress that participatory approaches to understanding heritage and its role in One Health should only be enacted in genuine partnership with interested community members, and never be imposed, to ensure that exchanges and outcomes are constructive and culturally appropriate.

Another important reflection of the workshop was that potential participatory or “bottom-up” approaches to One Health are currently constrained by contemporary systems of environmental and economic governance and restrictions on human rights to self-determination. For instance, a recurring theme of several discussions was the need to address the pervasive pressures of privatization, commercialization, and economic growth that underpin current global trends in environmental exploitation. Although some expressions of environmental heritage such as local fishing and hunting carry zoonotic disease risks and are sometimes unsustainable, overemphasis on such cases distracts attention from the inherent unsustainability and inequity of the capitalist world economy, and the roles of industrial food production, consumerism, and tourism in degrading ecosystems and elevating zoonotic disease risks. The idea of heritage offers hope because it embraces a vision of sustainable development that diverges from the dominant neoliberal conservation discourse, including rash calls to drastically expand and commodify protected areas.^3^ We contend that One Health outcomes will generally depend on systemic considerations embedded in heritage and underlying principles of social justice and environmental care, and the extent to which One Health policies rectify colonialism, pressures of capitalism and dominant classes, and upstream zoonotic disease risks in high-income settings.

In conclusion, the varied perspectives and case examples from this workshop suggest that integrating heritage into ecosystem management and zoonotic disease prevention could improve One Health outcomes. Recognizing the roles of Indigenous peoples and local communities in environmental conservation, and supporting communities in their conservation endeavors when and where support is welcomed, is particularly important to foster more balanced and reciprocal relationships between societies and the ecosystems on which they depend.^14^ Empowering communities to engage in environmental heritage practices is key because heritage is inextricably linked to local culture and crucial to generating and maintaining local knowledge and interests in environmental health. In many cases, this will require transformations of contemporary conservation laws that paradoxically inhibit the coexistence of humans and the rest of nature by restricting the customary institutions within which many long-enduring heritage practices are rooted.^15^ We therefore encourage scholars to advance One Health with further exchanges, reflections, and research on the critical role of everyday environmental heritage as both a reason and means for improving human, animal, and environmental health.

## Acknowledgments

We thank all participating villagers, Bertrand Laville, and all Lao scholars and students including Anouxay Pasitsone, Chansouk Vongsansouvanh, Kinnalone Salivong, Larnoy Xayyabouapha, Nim Pheephuk, Noudy Sengxeu, Sililack Youtthabouth, Sopha Keoinpaeng, Souphaphone Sorsavanh, Taiynoy Malaiphan, and Thippasurth Vongphachan, for guidance and inspiring exchanges that contributed to the development of the ideas encapsulated in this manuscript.

The workshop and the participation of its attendees were supported by the British Academy (ref. IOCRG\101013), the International Joint Laboratory PRESTO (PRotect-dEtect-STOp), the Embassy of France in Vientiane, and the Agence Universitaire de la Francophonie (AUF) in Laos.

## Author contributions

E.D. and M.J.H. led the organization of the workshop on which this article is based. All authors participated in the workshop, contributed to the original draft, and reviewed and approved the final draft.
